# Intravenous Dexmedetomidine-Ketamine Versus Ketamine-Propofol for Procedural Sedation in Adults Undergoing Short Surgical Procedures: A Randomized Controlled Trial

**DOI:** 10.7759/cureus.40676

**Published:** 2023-06-20

**Authors:** Anusha Kakarla, Laxman K Senapati, Asima Das, Mousumi Acharya, Sailaja Sukanya, Amit Pradhan

**Affiliations:** 1 Anaesthesia, Kalinga Institute of Medical Sciences, KIIT Deemed to be University, Bhubaneswar, IND; 2 Obstetrics & Gynaecology, Kalinga Institute of Medical Sciences, KIIT Deemed to be University, Bhubaneswar, IND

**Keywords:** propofol, ketamine, dexmedetomidine, sedation, rescue bolus, procedural interference

## Abstract

Background and objective

Moderate to deep sedation is a prerequisite during total intravenous anesthesia for short-duration surgeries, and it can be achieved by using individual drugs or in combination. Our study compared dexmedetomidine-ketamine (DK) versus ketamine-propofol (KP) in terms of sedation, procedural interference, hemodynamics, and incidence of side effects in patients undergoing short surgical procedures.

Methods

A total of 194 patients scheduled for short-duration elective surgeries were randomly allocated into two groups. Group DK received a loading dose of 1 µg/kg of dexmedetomidine and 1 mg/kg of ketamine followed by a maintenance infusion of dexmedetomidine at 0.3 µg/kg/h. Group KP received a loading dose of 1 mg/kg of ketamine and 1 mg/kg of propofol followed by a maintenance infusion of propofol at 25 µg/kg/h. For procedural interference, a rescue ketamine bolus was administered at 0.25 mg/kg. Patients were monitored for the requirement of rescue ketamine bolus, procedural interference, hemodynamics, sedation, recovery time, and adverse effects.

Results

The procedural interference was higher in group KP than in group DK and the difference was statistically significant (P=0.001). The time to the first rescue bolus was 8.72 ± 4.47 minutes in group KP and 10.82 ± 4.01 minutes in group DK, with a difference of 2.1 minutes (p*=*0.026). There was no statistically significant difference in the sedation scores between both groups except at time points of six minutes and 15 minutes.

Conclusion

For short-duration procedures, the DK combination is superior to the KP combination in terms of procedural interference and time to the first rescue bolus, while both groups were comparable with regard to safety and hemodynamics.

## Introduction

Over the past few decades, daycare surgical procedures have been on the rise. Daycare procedures help avoid a prolonged hospital stay. The concept of admission and discharge on the same day entails meticulous planning of anesthetic technique and hence helps in minimizing the undesired effects associated with surgery and anesthesia. Total intravenous anesthesia (TIVA) is defined as the use of intravenous agents for the induction and maintenance of anesthesia [1]. TIVA is preferred over endotracheal anesthesia for short surgeries requiring moderate to deep sedation because of its rapid induction and quicker recovery benefits [2]. Thus, faster recovery makes TIVA the suitable method of anesthesia for daycare procedures. For short-duration procedures, an ideal anesthetic drug should provide rapid and smooth induction, adequate analgesia, hemodynamic stability, and smooth and clear-headed recovery with minimal adverse effects [3]. Since the advent of TIVA, various anesthetics have been tried either as a sole agent or in combination. Pairing a hypnotic drug with an analgesic drug lends the advantage of covering both domains, facilitating smooth induction, pain-free maintenance, and quicker recovery.

Among the several drugs administered for procedural sedation, the combination of S-ketamine and propofol has been used extensively in operating rooms and emergencies [4]. Ketamine offers the advantages of analgesia, amnesia, and hypnosis without causing respiratory depression. Propofol is a quick-onset sedative-hypnotic drug that lacks analgesic properties [4]. Some of the untoward effects associated with propofol include dose-related cardiovascular and respiratory depression. Ketamine and propofol, when used in conjunction, lessen the side effects of each other, while the synergism of their hypnotic, sedative, and analgesic effects means that a lesser dose of each of them is required to achieve the desirable anesthetic effects [4].

Dexmedetomidine is a selective alpha-2 adrenergic receptor agonist drug with sedative, analgesic, and anxiolytic effects. Due to its notable advantages like hemodynamic stability, postoperative pain relief, and absence of respiratory depression, the combination of intravenous ketamine and dexmedetomidine is often utilized [5]. There are several studies in the literature on the combination of ketamine and dexmedetomidine in the pediatric population [6-12]. Ketamine and propofol have frequently been used in combination for procedural sedation. The objective of this study was to compare dexmedetomidine-ketamine (DK) with ketamine-propofol (KP) for procedural sedation in adult patients regarding the quality of sedation, hemodynamics, and safety profile.

## Materials and methods

Ethical consideration

This study obtained approval from the Institutional Ethical Committee, Kalinga Institute of Medical Sciences (KIMS) (KIIT/KIMS/IEC/412/2020). It was registered with the Clinical Trials Registry of India (CTRI/2021/09/036768).

Study design and eligibility criteria

This prospective randomized trial was conducted from September 2021 to August 2022 in the Department of Anesthesiology, KIMS, Bhubaneswar, Odisha after obtaining informed written consent from all patients. A total of 194 patients belonging to the American Society of Anesthesiologists (ASA) physical status I or II, aged between 18 and 65 years, and scheduled for elective daycare procedures of a duration of less than 30 minutes such as diagnostic hysteroscopy and endometrial biopsy (DHEB), polypectomy, suction and evacuation, transvaginal oocyte retrieval (TVOR), and testicular sperm aspiration (TESA) were enrolled for the study. The exclusion criteria were as follows: pregnancy, allergy to study drugs, ASA physical grade of III or IV, and obstructive sleep apnea.

Randomization and blinding

A computer-generated randomized sequence was generated and patients who fulfilled the inclusion criteria were randomly allocated into two groups of 97 each: Group DK and Group KP. Preanesthetic evaluation and laboratory investigations were done by adhering to institutional protocols. The study medications were prepared by personnel who did not participate in any other part of the study and were administered by an anesthesiologist who was not involved in the recording and analysis of data.

Drugs

The study drugs were prepared in identical 50-ml infusion syringes. The propofol infusion syringe contained 10 mg/ml propofol. The ketamine infusion syringes contained two different concentrations of ketamine diluted with normal saline (10 mg/ml and 1 mg/ml). The dexmedetomidine infusion syringe contained 100 µg dexmedetomidine diluted with normal saline (2 µg/ml).

Anesthetic management

On patients' arrival at the operating room, standard monitoring tools (electrocardiogram, pulse oximeter, and noninvasive blood pressure) were attached and the recording of heart rate (HR), blood pressure (BP), and oxygen saturation was started. Ringer lactate was infused after an 18-gauge IV cannula was inserted in the dorsum of the non-dominant hand. Based on the randomization, the subjects in Group KP received intravenous ketamine and propofol and the subjects in Group DK received intravenous dexmedetomidine and ketamine. Group KP patients received a loading dose of 1 mg/kg of propofol followed by ketamine at 1 mg/kg. For maintenance, propofol infusion was given at the rate of 25 mcg/kg/minute. Group DK patients received a loading dose of 1 µg/kg of dexmedetomidine over 10 minutes along with a loading dose of 1 mg/kg of ketamine. For maintenance, dexmedetomidine infusion was given at the rate of 0.3 µg/kg/hour. A rescue bolus dose of IV ketamine (0.25 mg/kg) was administered when needed to combat procedural interference. All subjects received supplemental oxygen via a face mask at the rate of 4 liter/minute.

At the end of the procedure, an injection of ondansetron (0.1 mg/kg) and a rectal suppository of diclofenac (100 mg) were given. We employed the injection of paracetamol 1 gm combined with the injection of tramadol (1.5 mg/kg) as standard postoperative analgesia.

Outcome measures

Hemodynamic parameters were recorded at baseline followed by every three-minute intervals till the end of the procedure. Any instance of bradycardia (HR less than 60 beats per minute) and hypotension (BP falling by at least 20% from baseline) was noted. The sedation score was measured with the Ramsay sedation scale [13]. The score was recorded at baseline, after induction, and at every three-minute interval till the end of the procedure. A minimum sedation score of 5 on the Ramsay sedation scale was considered adequate for procedural sedation. The time to the initial rescue bolus administration and the total number of doses of rescue bolus required for the surgery were noted. Other notable occurrences such as procedural interference (movement of lower limbs) and airway adverse events (apnea lasting >15 seconds) were also noted. Surgeon satisfaction and patient satisfaction were recorded by using a 3-point scale (1=satisfied; 2=ok; 3=dissatisfied). The non-verbal pain scale (NVPS) was used to evaluate procedural pain [14]. Any episodes of nausea and vomiting, recovery agitation, and recall of intraoperative events were documented in the postoperative period. Awakening time (time required to respond to auditory stimulus) and time to discharge from the post-anesthesia care unit (PACU) (Aldrete score of 9) was noted [15].

Sample size calculation

An interim analysis was conducted involving 20 patients to calculate the sample size. The number of rescue bolus was compared between the two groups. The proportion of subjects not requiring rescue bolus was 0.54 in group KP and 0.73 in group DK. Based on the data obtained, the sample size was calculated to be 194 (97 in each group) with an alpha (α) error of 0.05 and a power of 0.80.

Statistical analysis

The data were represented as mean ± standard deviation (SD) for continuous variables, whereas frequency and percentage were used to display categorical variables. The Chi-square or Fisher's exact test was used to assess the relationship between the two different groups. The continuous variables were evaluated using a student's t-test. The statistical analysis was carried out using IBM SPSS Statistics software version 26.0 (IBM Corp., Armonk, NY). A p-value of 0.05 or lower was considered statistically significant.

## Results

Study population

**Figure 1:**
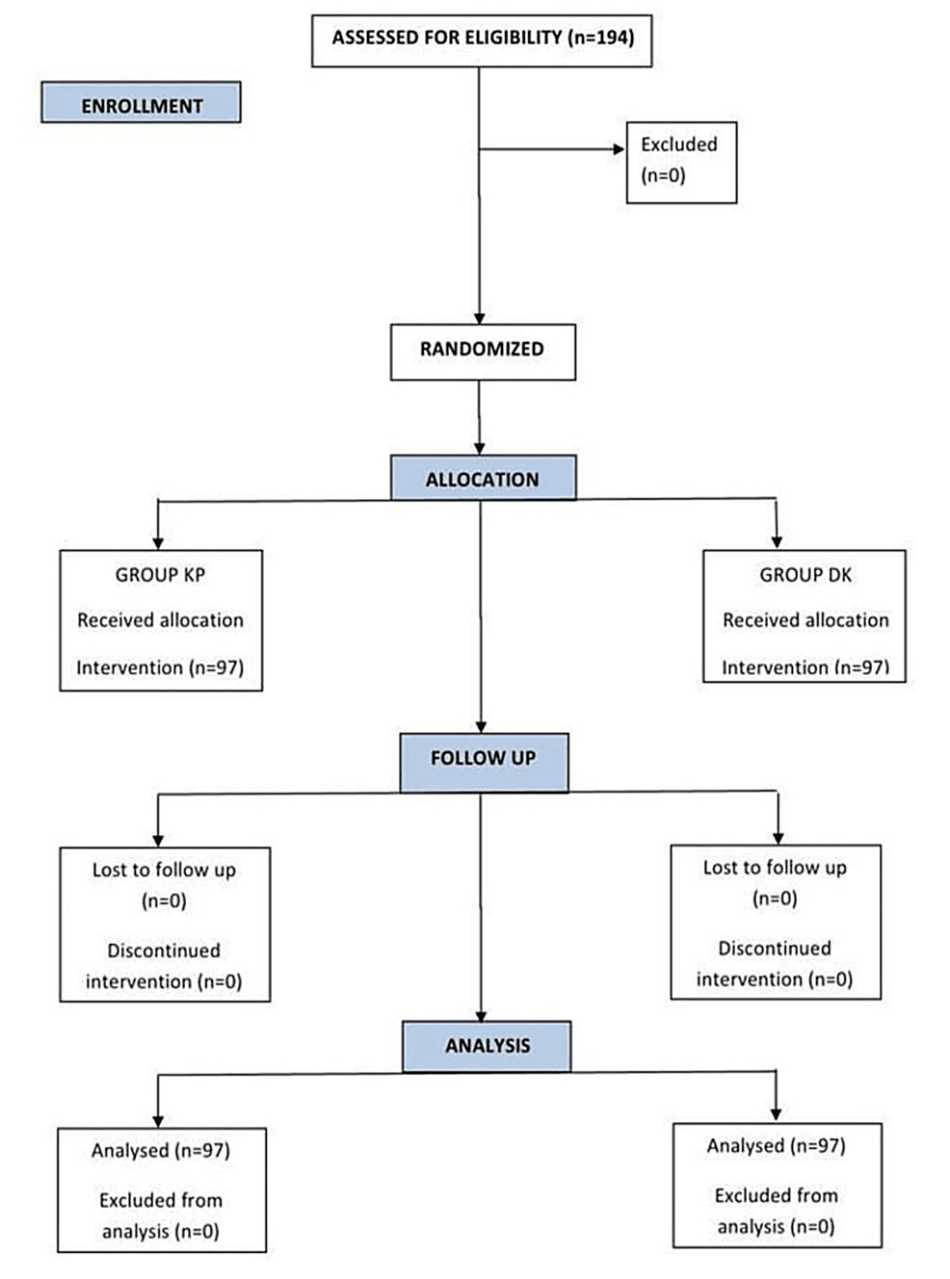
CONSORT flow diagram CONSORT: Consolidated Standards of Reporting Trials

The flow of patients through the trial is depicted in the Consolidated Standards of Reporting Trials (CONSORT) diagram (Figure 1).

The demographic characteristics, duration of the procedure, and distribution of ASA physical status I and II between the two groups were similar (Table 1). The difference between the groups regarding the type of procedure was statistically not significant (p=0.626).

**Table 1:** Demographic characteristics The data are presented as mean ± standard deviation KP: ketamine-propofol; DK: dexmedetomidine-ketamine; ASA PS: American Society of Anesthesiologists physical status

Characteristics	Group KP	Group DK	P-value
Age (years)	40.28 ± 9.52	39.35 ± 10.63	0.518
Weight (kg)	59.12 ± 9.60	60.75 ± 6.94	0.177
Duration (minutes)	17.39 ± 3.80	17.46 ± 4.97	0.909
ASA PS I:II	59:38	54:43	0.467

The primary and secondary outcomes

The primary outcome of the study was the number of rescue boluses of ketamine required during the procedure, and the difference between the groups with regard to this parameter was not statistically significant (p=0.822) (Table 2).

**Table 2:** Comparison of the primary outcome between the two groups The data are presented as mean ± standard deviation KP: ketamine-propofol; DK: dexmedetomidine-ketamine

Primary outcome	Group KP	Group DK	P-value
Number of rescue bolus	1.47 ± 0.70	1.44 ± 0.66	0.822

The time to first rescue bolus was significantly lower in the KP group compared to the DK group (p=0.026). The awakening time and recovery time were comparable in both groups (Table 3).

**Table 3:** Comparison of time-related outcomes between the groups The data are presented as mean ± standard deviation KP: ketamine-propofol; DK: dexmedetomidine-ketamine

Outcome	Group KP	Group DK	P-value
Time to first rescue bolus (minutes)	8.72 ± 4.47	10.82 ± 4.01	0.026
Awakening time (minutes)	4.47 ± 2.52	4.82 ± 2.58	0.340
Recovery time (minutes)	13.78 ± 6.50	13.96 ± 6.99	0.848

The procedural interference was analyzed using the Chi-square test, and the difference was statistically significant between the groups (p=0.001) (Table 4).

**Table 4:** Comparison of procedural interference between the groups The data are presented as numbers (%) and the Chi-square test was applied KP: ketamine-propofol; DK: dexmedetomidine-ketamine

	Group KP	Group DK	P-value
Procedural Interference	58 (59.79)	34 (35.05)	0.001

NVPS-revised was used to assess pain between two groups and the result based on the Chi-square was comparable (p=0.161) without any statistical significance. The surgeon satisfaction and patient satisfaction were analyzed with a Chi-square test and were comparable between the groups (p=0.405 and 0.828 respectively). The subjects in Group DK showed higher Ramsay sedation scores at six minutes and 15 minutes after induction, and the difference was statistically significant (Table 5).

**Table 5:** Comparison of Ramsay sedation scale score at different time intervals between the groups The student's t-test was used and data are presented as mean ± standard deviation KP: ketamine-propofol; DK: dexmedetomidine-ketamine; RSS: Ramsay sedation score

RSS	Group KP	Group DK	P-value
0 minute	1.01 ± 0.10	1 ± 0	0.318
3 minutes	5.93 ± 0.24	5.98 ± 0.10	0.054
6 minutes	5.85 ± 0.35	5.97 ± 0.14	0.001
9 minutes	5.84 ± 0.36	5.90 ± 0.29	0.221
12 minutes	5.83 ± 0.37	5.88 ± 0.31	0.325
15 minutes	5.82 ± 0.38	5.93 ± 0.24	0.019
18 minutes	5.91 ± 0.28	5.81 ± 0.39	0.175
21 minutes	5.60 ± 0.51	5.84 ± 0.37	0.199
24 minutes	6 ± 0	5.90 ± 0.22	0.409

Adverse outcomes

Occurrences of undesirable effects comprising nausea and vomiting, airway adverse events, and recovery agitation were comparable between the groups (Table 6). Recall of intraoperative events was not observed in any of the subjects.

**Table 6:** Comparison of safety outcomes between the groups The data arere represented as numbers (%). Chi-square and Fisher’s exact tests were applied KP: ketamine-propofol; DK: dexmedetomidine-ketamine

Outcomes	Group KP	Group DK	P-value
Airway adverse effects	2 (2.06)	1 (1.03)	0.561
Nausea and vomiting	2 (2.06)	4 (4.12)	0.407
Recovery agitation	3 (3.09)	3 (3.09)	1

Hemodynamic variables

**Figure 2:**
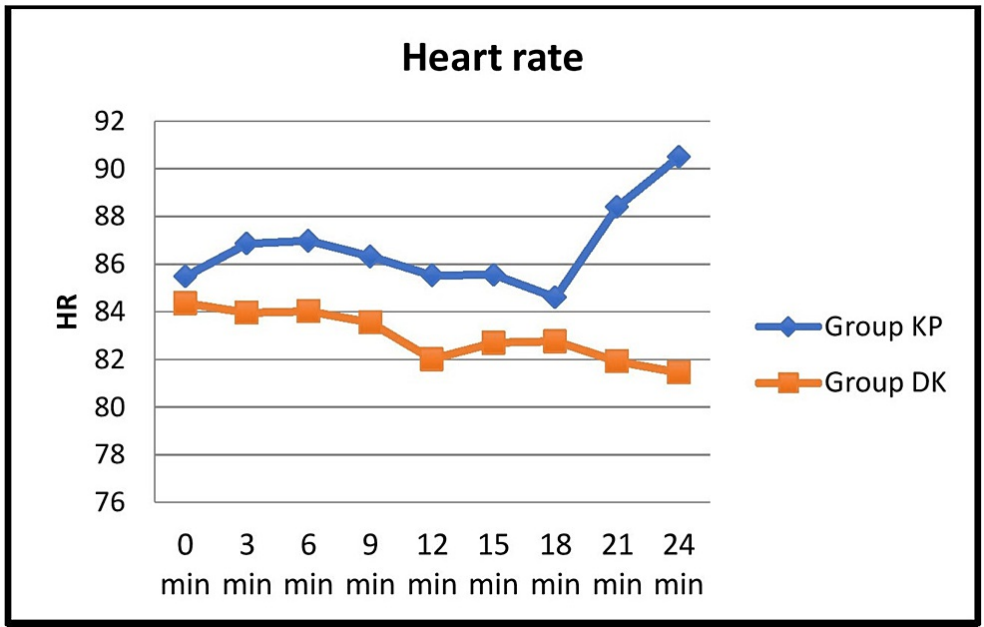
Comparison of the mean distribution of heart rate at 3-minute intervals between the two groups KP: ketamine-propofol; DK: dexmedetomidine-ketamine

The hemodynamic variables were recorded at three-minute intervals and evaluated using the student's t-test. No significant difference was seen when oxygen saturation was compared between the two groups. HR was comparatively lower in group DK, but the difference was statistically significant only at 12 minutes after induction (Figure 2).

**Figure 3:**
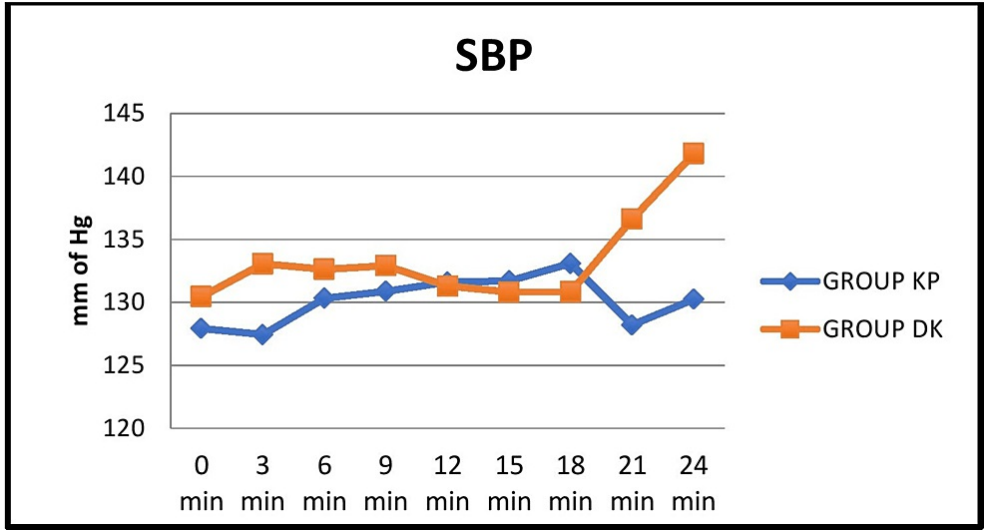
Comparison of the mean distribution of systolic blood pressure at 3-minute intervals between the two groups KP: ketamine-propofol; DK: dexmedetomidine-ketamine; SBP: systolic blood pressure

**Figure 4:**
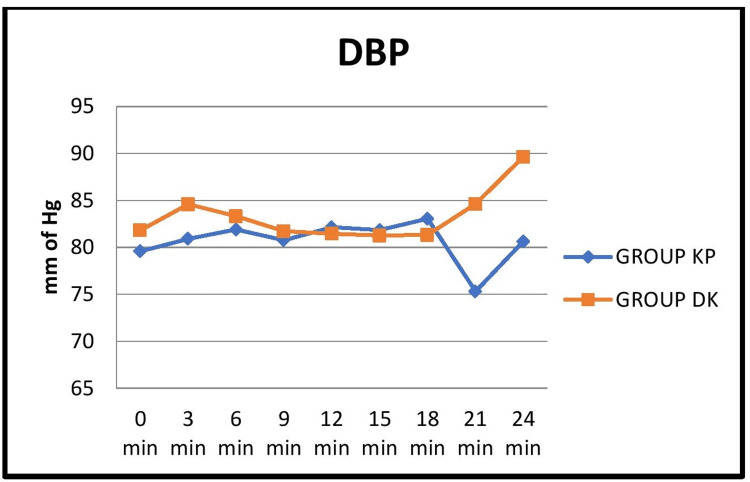
Comparison of the mean distribution of diastolic blood pressure at 3-minute intervals between the two groups KP: ketamine-propofol; DK: dexmedetomidine-ketamine; DBP: diastolic blood pressure

**Figure 5:**
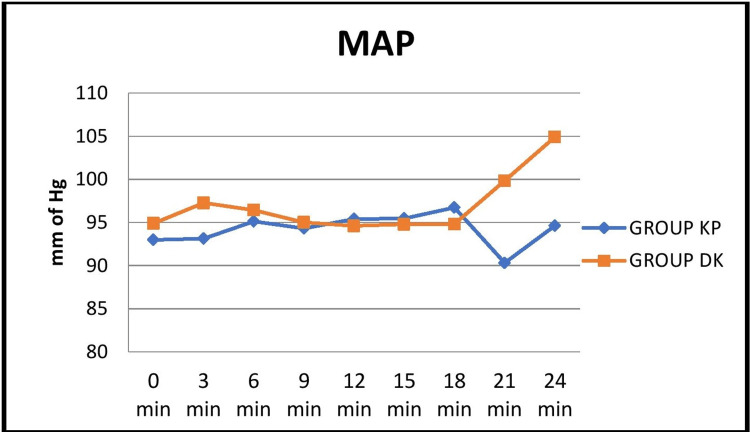
Comparison of the mean distribution of mean arterial blood pressure at 3-minute intervals between the two groups KP: ketamine-propofol; DK: dexmedetomidine-ketamine; MAP: mean arterial pressure

BP values were comparable between the groups except at three minutes after induction, where Group DK showed a significant increase in systolic, diastolic, and mean arterial pressures (Figures 3, 4, 5).

The respiratory rate (RR) was comparable between both groups, except at the time-point of 18 minutes when the DK group showed a significantly lower RR (p=0.048).

**Figure 6:**
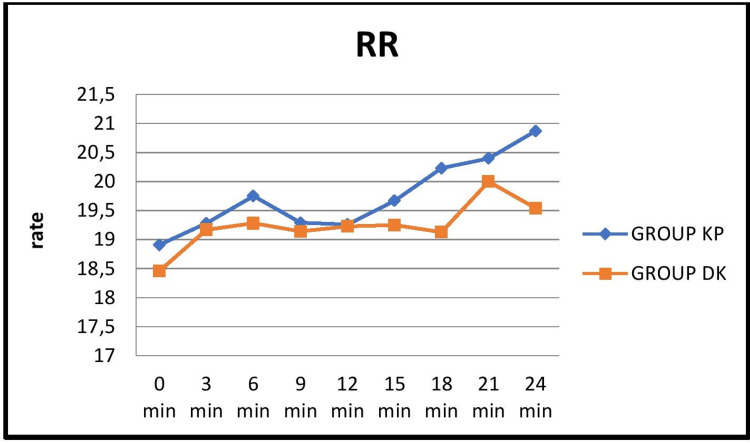
Comparison of the mean distribution of respiratory rate at 3-minute intervals between the two groups KP: ketamine-propofol; DK: dexmedetomidine-ketamine; RR: respiratory rate

## Discussion

Procedural sedation has been employed for a diverse range of procedures in both adult and pediatric age groups [7-12]. Over the last few decades, several studies have been conducted with various drugs with the aim of devising an ideal drug combination for procedural sedation [7-12]. This randomized study aimed to compare two different drug combinations - ketamine-propofol (KP) and dexmedetomidine-ketamine (DK) - in terms of the safety and efficacy of procedural sedation in elective short-duration surgeries in adults. The drug combination KP has been extensively studied in comparison to DK. The DK combination has been used in the pediatric age group for sedation in cardiac catheterization, tooth extraction, burn dressing, atrial septal defect (ASD) closure, and upper GI endoscopy [7-12].

Intraoperative analgesic supplementation

In terms of the primary outcome of our study, i.e., the number of rescue bolus of ketamine needed during the procedure, the results were comparable between the groups and had no statistically significant difference. Joshi et al. found that ketamine consumption was higher in the ketamine-dexmedetomidine group when compared with the ketamine-propofol group in pediatric patients during cardiac catheterization [7]. Similarly, Tosun et al. observed higher ketamine consumption in the ketamine-dexmedetomidine group compared to the ketamine-propofol group in children undergoing cardiac catheterization [6]. Algharabawy et al. found higher ketamine bolus consumption in the ketamine-dexmedetomidine group than the ketamine-propofol group in hepatic patients scheduled for upper GI endoscopy, but the difference was not statistically significant [16]. On the contrary, Amer et al., while performing sedation in pediatric endoscopy, found that the dexmedetomidine-ketamine combination showed a lesser need for additional drug supplementation [12]. In line with our finding, Canpolat et al. found no statistical significance with regard to the rescue bolus of ketamine [9,10]. However, in our study, it was observed that the incidence of procedural interference was higher in Group KP (p=0.001), and the difference was statistically significant. Hence, the total number of patients who required a rescue bolus of ketamine was higher in Group KP and the time to the first rescue bolus dose for procedural interference, if any, was longer in Group DK (p=0.026). In a study by El Sayed et al., patients who received the dexmedetomidine-ketamine combination had a longer time to the first rescue analgesia when compared to the fentanyl-ketamine group of patients [17], and this finding is in accordance with our study.

Recovery time

Our study showed that the time to eye-opening after the procedure was lower in Group KP (4.47 minutes), but the difference was not statistically significant. Subjects in both groups in our study showed quick awakening, which is a prerequisite for procedural sedation, and the difference between the groups was not statistically significant. Tosun et al., Joshi et al., and Amer et al. have observed longer recovery times in the dexmedetomidine group for cardiac catheterization and endoscopy procedures in pediatric patients [6,7,12]. Similarly, Goyal et al. observed that in adult patients scheduled for endoscopic retrograde cholangiopancreatography (ERCP), the recovery time was longer in the dexmedetomidine-ketamine group than in the propofol-fentanyl group [18]. Canoplat et al. observed longer recovery time with ketamine-dexmedetomidine than ketamine-propofol in pediatric patients undergoing burn dressing change [10]. Koruk et al. observed that recovery time was significantly shorter in the propofol-dexmedetomidine combination than in propofol-ketamine in pediatric patients undergoing transcatheter ASD closure [11]. Gopal et al. noted post-procedural recovery time to be significantly shorter in the dexmedetomidine-ketamine group compared to the dexmedetomidine-propofol group in patients scheduled for hysteroscopy [19].

In our study, the time required to reach Aldrete score ≥9 in PACU was 13.96 minutes in Group DK and 13.78 minutes in Group KP, and the difference was statistically insignificant (p=0.848); this finding is similar to results obtained from the study by Canpolat et al. on procedural sedation for pediatric tooth extraction [9]. The addition of ketamine to dexmedetomidine and propofol gives additional analgesic benefits [8,20]. Bingol Tanriverdi et al. observed lower post-procedure pain scores in patients who received dexmedetomidine compared to those who received propofol for hysteroscopy [21]. In our study, intraprocedural pain was comparable between the groups.

Patient and surgeon satisfaction

In our study, both groups were comparable in terms of patient and surgeon satisfaction. Sruthi et al. [22] and Ali Hassan [23] observed better surgeon satisfaction with ketofol than dexmedetomidine whereas Canpolat et al. [10] and Algharabawy et al. [16] observed no significant difference in terms of satisfaction. In the study by Canpolat et al. on tooth extraction, ketofol showed better surgeon satisfaction [9].

Intraoperative sedation

The sedation scores in our study were higher in Group DK at six minutes and 15 minutes after induction, and the difference was statistically significant. After 15 minutes post-induction, the level of sedation was comparable in both groups. Makwana et al. found that the mean sedation score in patients posted for upper limb surgeries under supraclavicular brachial plexus block was higher in the ketamine-dexmedetomidine group than the ketamine-propofol group throughout the intraoperative period, showing statistically significant p-values at 30, 40, 70, 80, 90, and 100 minutes [24].

Hemodynamic parameters

In our study, subjects in Group DK maintained lower HR when compared to Group KP, but statistical significance was appreciated only at 12 minutes post-induction. Dexmedetomidine decreases HR by decreasing norepinephrine levels [25]. A decrease in HR with dexmedetomidine was observed in many studies [6,7,16,21,22]. The studies by Canpolat et al. and Mogahd et al. showed no significance concerning changes in HR [9,26].

BP reading at three minutes after induction was found to be higher in group DK compared to group KP. The initial rise in BP can be attributed to the direct α2-mediated vasoconstrictive effect of dexmedetomidine. A biphasic response of hypertension followed by hypotension was observed by Park et al. in patients undergoing hysteroscopy with dexmedetomidine-remifentanil [27]. No episodes of hypotension were recorded in our study. After surgery, there was no incidence of hypotension and bradycardia in the DK group.

Adverse outcomes

In our study, oxygen saturation levels were maintained throughout the procedure in both groups without any episode of desaturation, which is consistent with the findings of Canpolat et al. [10]. Tosun et al. and Amer et al. recorded desaturation in pediatric patients who received a ketamine-propofol combination [6,12]. The intraprocedural respiratory rates were comparable in both of our groups except at the time point of 18 minutes after induction when Group KP showed a statistically significant higher RR compared to Group DK (p=0.048). 

In our study, two subjects in Group KP and four in Group DK experienced postoperative nausea and vomiting, but the difference between the groups in this regard was not statistically significant (p=0.407). However, in the study by Canpolat et al., the children who received ketamine-propofol for tooth extraction had lower incidences of nausea-vomiting compared to the ketamine-dexmedetomidine combination, and the difference was statistically significant [9]. The incidence of vomiting in adults receiving ketamine is reported to be 5-15% [28]. Because of the antiemetic property of propofol, the emetogenic potential of ketamine is counterbalanced, leading to a lower incidence of vomiting with the ketamine-propofol combination.

The incidence of airway adverse effects in our study was 2.06% in Group KP and 1.03% in Group DK, and the difference was not statistically significant (p=0.561). Propofol can cause apnea and respiratory depression and the addition of ketamine protects the airway by conserving airway reflexes and respiratory drive [20,29,30]. Dexmedetomidine is known to provide sedation with minimal respiratory depression, thereby lending the vital benefit of preserving respiratory function; hence, it is a suitable choice for procedural sedation [31,32].

In our study, the incidence of recovery agitation was similar in both groups with three subjects from each group experiencing it. Grégoire et al. studied the effect of the ketamine-dexmedetomidine combination in emergency settings and noticed incidences of recovery agitation [33]. The incidence of recovery agitation with ketamine is 10-20% in adults; however, with a ketamine-propofol combination for emergency procedural sedation, the incidence was 3.6% only [28,34]. Sruthi et al. did not observe any incidence of agitation with either dexmedetomidine or ketofol [22]. Tsai et al. observed recall among subjects undergoing fiberoptic nasotracheal intubation with dexmedetomidine infusion [35]. No subjects in our study had a recall of intraoperative events. Other side effects of ketamine, such as horizontal nystagmus, were not observed in any of our subjects.

Limitations

We utilized the Ramsay sedation scale to assess the degree of sedation. We believe that bi-spectral index (BIS) monitoring would not have been more helpful as it is not effective in assessing sedation under ketamine anesthesia.

## Conclusions

This prospective observational study showed that dexmedetomidine and ketamine as a combination is a superior alternative to the combination of ketamine and propofol for procedural sedation in adults undergoing short surgical procedures in terms of efficacy, safety, and hemodynamics. DK has an edge over KP on account of its added advantages such as lower procedural interference, longer time to the first rescue bolus, and better sedation. Therefore, the DK combination can be recommended as a safer and more efficacious choice for procedural sedation in short-duration surgical procedures.
